# Multiple Bilateral Parotid Calcifications in a Patient with Sjögren’s Syndrome: A Case Report

**DOI:** 10.30476/dentjods.2024.100095.2189

**Published:** 2024-12-01

**Authors:** Maryam Amirchaghmaghi, Mahrokh Imanimoghaddam, Atessa Pakfetrat, Maryam Bozorgi Baee

**Affiliations:** 1 Dept. of Oral and Maxillofacial Medicine, Oral and Maxillofacial Diseases Research Center, Mashhad University of Medical Sciences, Mashhad, Iran; 2 Dept. of Oral and Maxillofacial Radiology, Oral and Maxillofacial Diseases Research Center, Mashhad University of Medical Sciences, Mashhad, Iran; 3 Dept. of Oral and Maxillofacial Medicine, School of Dentistry, Alborz University of Medical Sciences, Karaj, Iran

**Keywords:** Sjögren's syndrome, Parotid gland, Calcification

## Abstract

This study investigates the calcifications in the parotid glands of a patient with Sjögren's syndrome (SS). A 52-year-old female patient presented for a routine dental examination who found to have multiple radiopacities in both parotid glands on panoramic radiograph. Further evaluation revealed swelling and tenderness in the parotid glands, decayed teeth, and dryness of the mouth and eyes. Ultrasound examination showed enlarged parotid glands with heterogeneous echogenicity, hypoechoic and cystic foci, and multiple calcifications. Laboratory tests indicated positive findings for rheumatoid factor and anti-SSA/SSB antibodies, consistent with a diagnosis of Sjögren's syndrome. Treatment primarily focuses on relieving symptoms and preventing clinical manifestations.

## Introduction

Sjögren's syndrome is a chronic autoimmune disease characterized by the destruction of exocrine glands, particularly salivary and lacrimal glands, resulting in dryness of the eyes and mouth [ 1 - 2 ]. Genetic factors, immunity, environmental factors, and epigenetics play a role in susceptibility to as well as progression of the disease, but its definitive etiology is not yet known. This condition predominantly affects women (with a 9 to 1 ratio compared to men) and has an average onset age of 50 years [ 2 - 3 ]. In the oral cavity, there is a decrease in saliva production, leading to dry, sticky, and erythematous oral mucosa. Additionally, atrophic filamentous papillae are observed on the dorsum of the tongue [ 3 ]. 

Parotid gland calcifications, occurring in 0.1% to 1% of the global population, can develop in the parenchyma or ducts of the salivary glands [ 4 - 6 ]. Previous studies have described various pathological conditions associated with parotid gland calcifications, including autoimmune, inflammatory, infectious, and neoplastic conditions. The proposed association between parotid gland calcifications and chronic inflammatory conditions suggests that chronic destruction of the exocrine glands by lymphocytes eventually leads to intraductal calcifications [ 7 ]. Calcifications located in the parenchyma of the parotid gland are less common than those within the ducts and are usually round and uniform in size [ 8 ]. Parotid gland calcifications have been recently reported as a new and rare feature of Sjögren's syndrome, and for its investigation, dental panoramic radiography, ultrasonography, sialography, or cone beam computed tomography (CBCT) are utilized [ 9 ]. However, when parotid gland calcifications are incidentally detected on computed tomography (CT) imaging, the patient's medical history often does not reveal any infectious, inflammatory, or neoplastic background. Therefore, the presence and clinical significance of incidental parotid gland calcifications in asymptomatic patients remain unclear [ 7 ]. The presence of scattered, punctate, cavitated sialoadenitis without evidence of obstruction strongly indicates the diagnosis of Sjögren's syndrome and is also useful in the staging of the disease [ 10 ].

In this study, we present the case of a patient who presented with multiple bilateral parotid gland calcifications on a panoramic radiograph and was eventually diagnosed with Sjögren's syndrome following further investigations.

## Case Presentation

**Figure 1:**
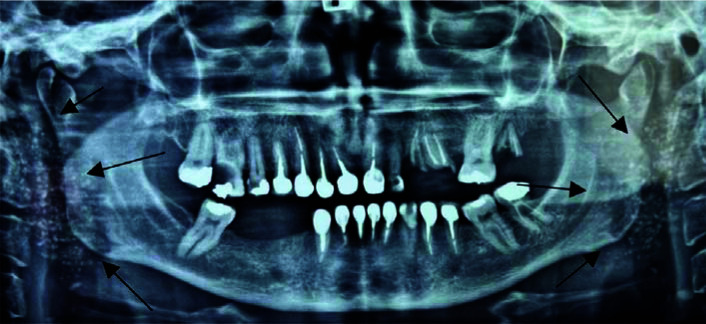
The panoramic image shows multiple dental caries and multiple globular radiopacities behind the mandibular ramus on both sides associated with the parotid gland

A 52-year-old female patient presented to a dentist for routine dental examination. While a panoramic radiograph was provided to assess the dental condition, the dentist noticed multiple radiopacities in both parotid glands (Figure 1). Hence, the patient was referred to the Department of Oral and Maxillofacial Diseases at Mashhad Dental School for further evaluation. The patient had no significant medical history except for migraines, for which she used acetaminophen for treatment. There were no positive findings in the family history. External oral examination revealed swelling of the salivary glands, facial asymmetry, tenderness to touch in both parotid glands, and no palpable abnormal lymph nodes.

**Figure 2:**
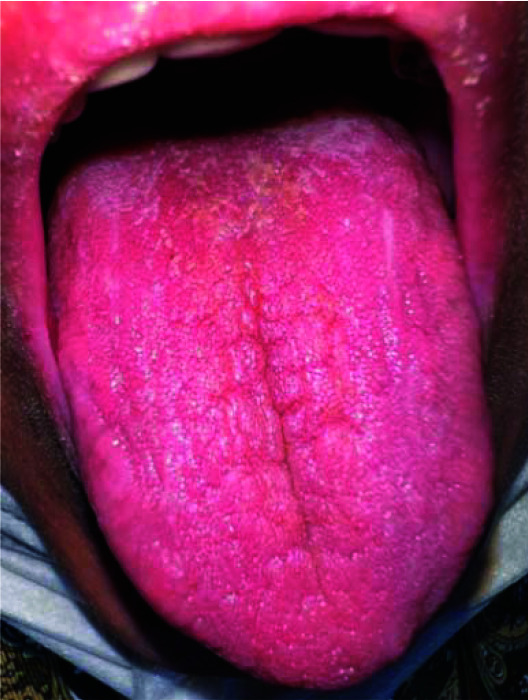
Erythematous and atrophic areas on the dorsum of the tongue

Intraoral examination revealed several decayed teeth. Milked saliva from both parotid glands appeared clear, but the amount of salivary flow from the Stensen's duct on the left side was slightly lower than the right side. Saliva was thick in both sides. No mucosal lesions were observed in other areas of the mouth, but erythematous and atrophic areas were seen on the dorsum of the tongue (Figure 2). The patient reported experiencing dryness of the mouth and eyes for the past 7-8 months.

The panoramic view showed multiple dental caries and multiple globular radiopacities behind the mandibular ramus on both sides, associated with the parotid salivary gland (Figure 1). Sjögren's syndrome was suggested as a probable diagnosis due to the presence of multiple calcifications in both parotid glands and the history of dry mouth and dry eyes.

Further investigations, including ultrasound examination of the bilateral parotid glands, were requested. The results showed that the parotid glands were larger than normal, with heterogeneous echogenicity and numerous hypoechoic and cystic foci, along with multiple calcifications in the tissue of the bilateral parotid glands.

Additional tests were requested to evaluate the possibility of Sjögren's syndrome. In the complete blood count (CBC), the patient showed only a decrease in mean corpuscular volume (MCV) and mean corpuscular hemoglobin (MCH) and an increase in red cell distribution width (RDW-CV). In the antinuclear antibody (ANA) profile tests, rheumatoid factor (RF:366), anti-RO/SSA (60 kDa), anti-RO/SSA (52 kDa), and anti-SSB (anti La) were positive. Based on the criteria set by the 2016 American College of Rheumatology, the rheumatologist confirmed the diagnosis of Sjögren's syndrome, and no minor salivary gland biopsy was requested. Treatment was initiated with hydroxychloroquine 200mg every 12 hours and prednisolone 5mg/ day.

During the 3-month follow-up, the patient showed improvement in dryness of the eyes and mouth. Additionally, a fine-needle aspiration (FNA) was requested under sonographic guidance to rule out mucosa-assisted lymphoid tissue (MALT) lymphoma [ 11 ], but the biopsy showed sections of parotid gland tissue with severe acinar atrophy and lymphoplasmacytic infiltration, and no evidence of lymphoproliferative lesions. The patient was continuously under the medication (Hydroxychloroquine 200 mg, Prednisolone 5 mg) and follow-up of the rheumatology specialist, and during the 6-month follow-up, the symptoms of dry eyes and dry mouth have improved with no specific complaints reported by the patient.

## Discussion

Sjögren's syndrome is an autoimmune disease that progresses slowly and can affect not only the exocrine glands but also other organs in the body, including the joints, gastrointestinal system, and central nervous system, and it can also increase the risk of lymphoma [ 12 ]. There are two forms of this disease including primary Sjögren's syndrome when the disease occurs alone, and secondary Sjögren's syndrome, which is associated with other autoimmune diseases [ 13 ]. The classification criteria for Sjögren's syndrome (2016) are based on five items: histology, immunology, two ophthalmological tests, and salivary flow evaluation [ 14 ].

Recently, the occurrence of multiple calcifications in the parotid gland has attracted attention as a potential effective indicator for diagnosing Sjögren's syndrome [ 1 ]. Although the mechanism of their formation in Sjögren's disease is not fully understood [ 11 ], it is hypothesized that these calcifications are related to altered salivary flow and high concentrations of calcium salts in the saliva. Parotid calcifications seen on imaging are usually attributed to chronic autoimmune/inflammatory conditions [ 7 ]. Additionally, chronic inflammation resulting from chronic destruction of exocrine glands by lymphocytes can lead to dysplastic calcifications in Salivary gland tissues and ducts [ 6 ].

Active salivary lobules that showed decreased soft tissue attenuation compared to adipose tissue were noted to decrease, resulting in a heterogeneous parotid gland in Sjögren's syndrome patients [ 10 ]. In our study, the bilateral parotid salivary glands were larger than normal, with heterogeneous echogenicity and numerous small hypoechoic and cystic areas, along with multiple calcification foci visible in the tissue of bilateral parotid glands.

In a study by Masahiro Izumi *et al*. [ 6 ], it was reported that these calcifications were regular and round in shape. Clinical symptoms were minimal in cases with calcifications. Although reduced salivary flow rate was associated with the occurrence of sialolithiasis, the severity of damaged parenchyma of the parotid gland did not correlate with the presence of calcifications in Sjögren's syndrome patients [ 6 ]. In our study, both bilateral parotid salivary glands had clear saliva, and the amount of saliva flow from the Stensen's duct on the left side was slightly lower than the right side. The saliva in both sides appeared thick. 

The heterogeneity of parotid glands in patients with Sjögren's syndrome, using ultrasound and magnetic resonance imaging (MRI), was investigated [ 15 - 17 ]. The parotid glands in Sjögren's syndrome patients were found to be heterogeneous, consisting of areas of low and high signal intensity on T1-weighted MR images [ 17 - 18 ]. This heterogeneity can also be observed in ultrasound studies, which have been proven to be a non-invasive and accurate tool for diagnosing Sjögren’s syndrome [ 18 ]. Several hypoechoic regions within the glands can be identified, and heterogeneous glands were found gradually in patients with advanced disease [ 19 ]. In this study, we also asked for additional ultrasound examinations of the parotid on both sides. 

CT scans have not been routinely included in suspected or confirmed Sjögren’s syndrome cases due to its inherent radiation effects. However, CT is widely used in head and neck imaging for evaluating parotid gland diseases. A better understanding of CT manifestations of Sjögren’s syndrome is necessary for accurate differential diagnosis. 

San *et al*. [ 10 ] reported that the occurrence of calcifications in bilateral parotid glands in CT examinations of Sjögren’s syndrome patients was 29.4-35.2%. Most radiological examinations for Sjögren’s syndrome include sialography, which are less effective in diagnosing small and weak calcifications. If CT examinations are performed more frequently, the potential presence of calcifications in the parotid glands may be evident in many Sjögren’s syndrome patients.

The pathological evidence of Sjögren's syndrome is still not precisely determined because surgical biopsy of the parotid glands is not common in Sjögren’s syndrome patients. Existing pathological knowledge about Sjögren’s syndrome is mainly derived from minor salivary gland biopsies, which have a completely different lobular structure from the parotid glands [ 10 ]. However, in this study, no request was made for minor salivary gland biopsies.

The causes and pathological associations of parotid gland calcifications based on previous studies include autoimmune, inflammatory, infectious, and neoplastic conditions [ 6 ]. In a study conducted by Karen Buch *et al*. [ 7 ], parotid gland calcifications were associated with HIV infection, alcohol addiction, chronic kidney disease, autoimmune diseases, and elevated alkaline phosphatase. Autoimmune diseases accounted for 11% of Sjögren’s syndrome patients with parotid gland calcifications. In patients with bilateral multiple calcifications within the parotid parenchyma, autoimmune diseases and inflammatory parotitis should be considered.

In addition to CT scans, panoramic radiography, commonly available in dental clinics, can be a useful tool in aiding the diagnosis of Sjögren's syndrome. Similar to our report, in another case by Kondo *et al*. [ 1 ], a patient with dental complaint have been presented with multiple parotid gland calcifications incidentally found on panoramic radiography, which confirmed the diagnosis of Sjögren's syndrome after further investigations. Other case reports describe patients who initially sought medical attention due to unilateral or bilateral parotid gland pain and swelling, with multiple bilateral parotid gland calcifications identified on subsequent paraclinical examinations, leading to the diagnosis of Sjögren's syndrome [ 4 , 10 ].

Currently, the treatment of Sjögren's syndrome mainly focuses on symptomatic relief and prevention of clinical manifestations. It includes saliva substitutes, topical eye products like artificial tears, oral muscarinic antagonists, hydroxychloroquine, glucocorticoids, immunosuppressive agents, and biologic therapies [ 9 , 20 ]. However, considering the absence of symptoms in our patient and the controlled management with medical treatment, additional interventions to remove radio opaque bodies were not prescribed. More studies need to be conducted to determine the percentage of patients with Sjögren’s syndrome in whom this appearance is visible on panoramic radiographs, CT scans, or sonography. It also needs to be determined whether it has diagnostic value or not.

To report this case, informed consent was obtained from patient.

## Conclusion

This study highlights the heterogeneity and calcifications in the parotid glands of patients with Sjögren's syndrome. Ultrasound and MRI examinations provide valuable insights, and CT scans may also be beneficial for differential diagnosis and detecting calcifications. Panoramic radiography can be a useful tool in dental clinics for early detection of Sjögren's syndrome. Treatment primarily focuses on relieving symptoms and preventing clinical manifestations. Further research and investigations are needed to gain a better understanding of the pathological aspects and optimize the management of Sjögren's syndrome.
